# The Central Effects of Botulinum Toxin in Dystonia and Spasticity

**DOI:** 10.3390/toxins13020155

**Published:** 2021-02-17

**Authors:** Pavel Hok, Tomáš Veverka, Petr Hluštík, Martin Nevrlý, Petr Kaňovský

**Affiliations:** Department of Neurology, Faculty of Medicine and Dentistry, University Hospital Olomouc, Palacký University Olomouc, 779 00 Olomouc, Czech Republic; pavel.hok@fnol.cz (P.H.); phlustik@upol.cz (P.H.); martin.nevrly@fnol.cz (M.N.); petr.kanovsky@fnol.cz (P.K.)

**Keywords:** dystonia, spasticity, botulinum toxin, electrophysiology, functional magnetic resonance imaging, neural plasticity

## Abstract

In dystonic and spastic movement disorders, however different in their pathophysiological mechanisms, a similar impairment of sensorimotor control with special emphasis on afferentation is assumed. Peripheral intervention on afferent inputs evokes plastic changes within the central sensorimotor system. Intramuscular application of botulinum toxin type A (BoNT-A) is a standard evidence-based treatment for both conditions. Apart from its peripheral action on muscle spindles, a growing body of evidence suggests that BoNT-A effects could also be mediated by changes at the central level including cerebral cortex. We review recent studies employing electrophysiology and neuroimaging to investigate how intramuscular application of BoNT-A influences cortical reorganization. Based on such data, BoNT-A becomes gradually accepted as a promising tool to correct the maladaptive plastic changes within the sensorimotor cortex. In summary, electrophysiology and especially neuroimaging studies with BoNT-A further our understanding of pathophysiology underlying dystonic and spastic movement disorders and may consequently help develop novel treatment strategies based on neural plasticity.

## 1. Introduction

Therapy with botulinum toxin type A (BoNT-A) is currently used in a wide range of medical conditions, including disorders characterised by muscle hyperactivity. Following an intramuscular application, primary action of BoNT-A occurs at the neuromuscular junction: BoNT-A enters presynaptic terminals and acts as a metalloproteinase by cleaving the components of the soluble N-ethylmaleimide-sensitive factor attachment protein (SNAP) receptor (SNARE) complex, effectively blocking acetylcholine release and neuromuscular transmission [1]. This peripheral effect is transient although long-lasting. It has a gradual onset as maximum changes at the synapses can be observed at approx. 4 weeks after application and slowly wears off as the neuromuscular junction recovers within 12 weeks [2,3]. Thanks to the long-lasting effect and good safety profile, intramuscular injections of BoNT-A have become the first-line treatment for focal spasticity [4,5,6] and dystonia [7]. Although, in general, clinical improvement usually follows the time course of the peripheral changes, there are a number of reports of discrepancies between the clinical symptoms and the duration or degree of neuromuscular junction blockade [3]. Clinical observations and an increasing number of neurophysiological and neuroimaging studies suggest the central (remote) effects of BoNT-A injected in the periphery. The theory of retrograde transport, although supported by several animal studies in particular [8], remains controversial in human use of BoNT-A at therapeutic dosing [9]. While the BoNT-A molecules transported via peripheral nerves may act on the spinal cord, the direct impact on distant central structures is rather hypothetical. The central effects are thus more likely elicited by indirect mechanisms that involve reduced afferentation from muscles treated with BoNT-A. In light of the fact that BoNT-A hampers the neuromuscular transmission between intrafusal muscle fibres and gamma-motoneuronal endings, it inevitably alters the afferentation from muscle spindles via the Ia afferents [10,11]. Reduced excitability of the spinal pathways thus indirectly modulates supraspinal motor control centres including the sensorimotor cortex [11]. This is the presumed mechanism for how BoNT-A injected in the periphery may induce cortical reorganization [11]. Based on this hypothesis, it has been proposed that some of the clinical BoNT-A effects could be mediated (indirectly) by central structures [10,11,12].

Although they are distinct conditions, dystonia and spasticity share common features such as involuntary movements, spasms, abnormal posturing and impaired movement performance resulting from muscle hyperactivity and co-contractions. By definition, dystonia manifests with sustained or intermittent muscle contractions which lead to abnormal posturing and involuntary movements [13]. Spasticity is a disorder of sensorimotor control, resulting from an upper motor neurone lesion, manifesting as intermittent or sustained involuntary activation of muscles [14]. Both dystonia and spasticity, despite some substantial differences, are associated with disordered sensorimotor control (maladaptive changes). These central alterations observed in both conditions may be modified by modulation of the somatosensory afferents. The reduction of pathological input from the Ia afferent fibres caused by BoNT-A can therefore induce cortical reorganization (adaptive plasticity). From this perspective, BoNT-A possibly acts as a central-modulating agent that promotes neuroplasticity in a beneficial way; whether it can induce a lasting change in the nature of the discussed diseases remains a question.

Human research of neuroplasticity is essentially limited to non-invasive or semi-invasive methods. Electrophysiology can provide superb temporal resolution down to milliseconds, whereas modern neuroimaging methods, including functional magnetic resonance imaging (fMRI), can provide outstanding spatial resolution. Combined electrophysiological and fMRI evidence may thus provide a more complete picture of the central sequelae of BoNT-A therapy. Hence, we review evidence for the central effects of BoNT-A in dystonia and spasticity from both electrophysiology and functional neuroimaging studies, in an attempt to provide a comprehensive overview of the mechanisms triggered by BoNT-A that may possibly mediate its clinical effect. Although the central effects of intramuscular BoNT-A have been summarised in a comprehensive review by Weise et al. [3], here, we add other pieces of recent evidence, scrutinise the methodology of the included studies, and provide our own interpretation of the available data.

Due to a considerable complexity of the neuroimaging and electrophysiological methods referred to in this review, a brief glossary is provided in Table 1.

## 2. Cortical Sensorimotor Plasticity Due to Reduction in Sensory Input

Cortical motor representations are not only subject to change in response to enhanced afferentation or practice, but also due to sensory loss. Cortical reorganisation with increased cortical excitability and decreased intracortical inhibition was observed in upper- and lower-limb amputees [20,21,22,23], i.e., changes similar to the effects of some plasticity-inducing peripheral stimulation protocols such as paired associative stimulation (PAS) [24]. Similar changes have been observed following reversible means of deafferentation. During ischaemic nerve block (INB), the intracortical GABA-ergic inhibitory influence decreases proximal to the block, thus, cortical excitability and a readiness for plastic changes are increased [23,25,26,27]. The same was observed during pharmacologically induced regional anaesthesia [28]. The effects of INB can be further facilitated by muscle practice [29] or repetitive transcranial magnetic stimulation (rTMS) [26,27]. A similar effect can also be elicited in distal hand muscles with improvement of skilled performance when the experiment is inverted, and an anaesthetic drug is applied to induce upper arm anaesthesia in chronic stroke patients [30]. Analogous effects have also been demonstrated in homotopic cortical regions of the limb contralateral to the deafferented extremity, which has been associated with decreased interhemispheric inhibition (IHI) [31]. Furthermore, anaesthesia of a healthy arm was shown to improve skilled motor performance of a paretic arm in patients after stroke [32].

Opposite changes may also be observed, however, in the limb parts deprived of somatosensation. Cortical representations of hand muscles are reduced when pure cutaneous sensory loss is achieved around the particular muscles using nerve anaesthesia while the muscle afferentation is spared [33]. Reduction of sensory input with concomitant decrease of muscle use due to immobilisation also diminishes the cortical representation of the muscle [34].

In general, sensory deprivation increases cortical excitability and promotes the plasticity of the adjoining non-deprived muscles, as well as in contralateral limbs. In contrast, the muscles in the deafferented segment show reduced cortical excitability.

## 3. Selective Muscle Denervation: Botulinum Neurotoxin (BoNT-A)

Apart from rather non-selective deafferentation using INB or anaesthetic drugs, afferentation can be selectively reduced from muscles using BoNT-A, leading to central sensorimotor adaptive changes, as described in the Introduction. In the following sections, we first discuss electrophysiological evidence, followed by functional neuroimaging data.

### 3.1. Electrophysiological Evidence for the Central Effects of BoNT-A

#### 3.1.1. Healthy Subjects

The electrophysiological evidence in healthy subjects is scarce [35]. The only study using transcranial magnetic stimulation (TMS) by Kim et al. [36] evaluated cortical excitability in 10 healthy subjects following BoNT-A application into the *extensor digitorum brevis* muscle. The authors reported increased short-interval intracortical inhibition (SICI), decreased intracortical facilitation (ICF) and significant shortening of cortical silent period (CSP). Notably, these changes were present 1 month after injection and were maintained for at least 3 months [36]. Much more data on the central effects of BoNT-A is, however, available from clinical studies in patient cohorts, in which BoNT-A is a recommended treatment, such as dystonia and spasticity.

#### 3.1.2. Dystonia

It has been long established that patients with dystonia exhibit abnormal sensory processing of muscle spindle afferentation [37,38] and there is compelling evidence that BoNT-A injections may normalise some of those findings. Most of the findings, however, stem from studies on different forms of focal dystonias, which limits the generalizability of results. Abnormal sensorimotor integration in the cervical dystonia was demonstrated, for example, in the precentral P22/N30 component of the median nerve somatosensory evoked potentials (SEP). Patients specifically exhibited higher P22/N30 amplitude in the side contralateral to the involuntary head rotation, as compared to both the ipsilateral side and to healthy controls [18,39]. Following BoNT-A treatment, the amplitude of P22/N30 was reduced to normal levels [39]. Neither baseline SEP abnormalities nor treatment-related changes were subsequently observed, however, in patients with focal hand dystonia, suggesting that various forms of dystonia may involve distinct pathophysiological mechanisms and responses to therapy [40].

Further studies indicated that dystonia might be associated with additional electrophysiological abnormalities confined to the motor cortex, including changes affecting inhibitory circuits. In fact, the previously reported abnormal augmentation of P22/N30 was associated with decreased SICI in the same hemisphere [41] which also improved after BoNT-A [42]. Decreased SICI was also demonstrated in 15 patients with focal hand dystonia [43] and in a mixed cohort of 12 patients, the majority which had generalised forms of dystonia [44]. Although Ridding et al. [43] did not assess the treatment effect, Gilio et al. [44] observed that SICI transiently normalised after BoNT-A. A later study in a smaller, but more homogeneous group of 6 patients with focal hand dystonia found, however, no evidence of any treatment-related changes in intracortical inhibition despite the reduced peripheral response [45]. Similarly, no such changes were reported in a group of 10 patients with blepharospasm [46]. A study in a mixed (BoNT-A naïve and chronically treated) cohort of 10 patients with adductor-type spasmodic dysphonia showed that patients had higher motor evoked potentials (MEP) amplitude during speech production and, on the whole, shorter CSP in the dominant hemisphere before treatment. The treatment with BoNT-A led to a decrease in MEP amplitude, but the effect of treatment on CSP was not tested [47].

The variability of treatment-related effects might be related either to differences among the phenotypes of dystonia or to the relatively low sample sizes. Even when the overall MEP amplitude remains unchanged, there can still be treatment-related changes in cortical organisation, as indicated by a series of studies demonstrating shifts and distortions of cortical motor maps in patients with cervical or focal hand dystonia and their temporary normalisation after BoNT-A [48,49,50].

Additional evidence for the central effects of BoNT-A originates from studies that evaluated how the treatment interferes with the processing of muscle vibration. A study by Trompetto et al. [51] demonstrated that BoNT-A treatment in writer’s cramp patients reduced otherwise normal tonic vibration reflex (TVR) and decreased peripheral response (maximum M-wave amplitude) from the injected muscle. Longitudinal evaluation in 2 patients revealed that persisting clinical effects were still associated with decreased TVR despite a normalised M-wave. It was proposed that reduced afferent inflow from the intrafusal fibres and subsequent defective muscle reinervation are responsible for the persisting treatment effects [51]. We also speculate that the outlasting BoNT-A effect might represent plastic changes interacting with the supraspinal component of the TVR [52] directly at the central level. Similarly, BoNT-A injection into the affected sternocleidomastoid muscle in patients with cervical dystonia prevented facilitation of the MEP amplitude during muscle vibration [53], which is also considered to be of cortical origin [54,55]. The effect was temporary as it returned partially to baseline after the expiration of the BoNT-A blockade. Notably, in comparison with healthy control subjects, vibration-induced MEP facilitation in the patient group was already moderately attenuated at baseline, suggesting a possible carry-over effect from previous injections in pre-treated patients [53].

Apart from affecting the immediate responses to peripheral stimulation, BoNT-A was also shown to prevent abnormal plasticity triggered by peripheral stimulation in patients with focal dystonia [56,57,58,59]. In untreated patients with dystonia, both facilitatory and inhibitory protocols of PAS cause changes in cortical excitability that spread beyond the somatotopic representations of the stimulated sites and are more diffuse than in healthy controls. These changes were observed in various forms of focal dystonia, including cervical dystonia, blepharospasm, and writer’s cramp, suggesting more global abnormalities in the processes mediating the long-term potentiation (LTP)-like and long-term depression (LTD)-like plasticity [56,57]. The application of BoNT-A in cervical dystonia patients abolished this facilitatory effect of PAS completely after 1 month. This BoNT-A effect was also temporary since the facilitation after PAS was partially restored after a 3-month follow-up [58]. A recent study in 16 cervical dystonia patients chronically receiving BoNT-A with suboptimal outcome showed that PAS-evoked plasticity was only reduced in patients who underwent BoNT-A administration combined with physiotherapy. The reduction of PAS-evoked plasticity correlated with clinical scores (TWSTRS severity and pain measure). The study reported, however, no detailed numerical values of PAS and TMS parameters other than relative change to baseline to allow for a full critical evaluation of the study conclusions regarding PAS-evoked plasticity [59].

#### 3.1.3. Spasticity

The available literature on central effects of BoNT-A in spasticity is more limited than in dystonia. Several studies have reported, however, that apart from alleviation of spasticity, BoNT-A also improved abnormal SEP [60,61,62]. In general, spasticity was associated with decreased SEP amplitude that increased after treatment, which is quite opposite to the effect reported in dystonia [39]. Recent data from our lab in a large cohort of 30 patients show that, despite confirming decreased SEP at baseline, BoNT-A did not lead to any changes in SEP amplitude throughout a 3-month follow-up [63]. Whereas SEPs seem to be affected differently in spasticity and dystonia, the processing of proprioceptive (vibratory) stimuli appears to be similarly altered by BoNT-A in both conditions. BoNT-A was shown to decrease the TVR amplitude in spastic [64], as well as dystonic muscles [51] and both effects outlasted the peripheral effects of BoNT-A [51,64].

To our knowledge, only two TMS studies evaluated motor cortical excitability following BoNT-A treatment in spasticity disorders: one assessed BoNT-A in lower limb spasticity [65] and one in upper limb spasticity [66]. Pauri et al. [65] observed increased MEP latency and central conduction time following BoNT-A application into the shank muscles in patients with paraparesis. There was no change, however, in MEP amplitude that would suggest any supraspinal effect. Redman et al. [66] evaluated, in contrast, the shift in cortical representation of the first dorsal interosseus muscle in children with cerebral palsy following BoNT-A but found no statistical difference in comparison with the controls. This illustrates the paucity of direct evidence of motor cortex involvement in the therapeutic effects of BoNT-A (for a review of other neurophysiological techniques, see [67]).

In summary, electrophysiological data available to date provide some indications that BoNT-A affects cortical motor representations and somatosensory processing, similar to the experimental procedures reducing the afferentation in a more diffuse way (e.g., INB). The literature is still too scarce, however, to draw any definite conclusions and several controversies, such as the presence of BoNT-A-related changes in SICI or SEP, are yet to be resolved.

### 3.2. Neuroimaging Evidence for Central Effects of BoNT-A

Similar to electrophysiology research, imaging studies of central effects of BoNT-A in healthy subjects (e.g., as a control group) are virtually non-existent. Numerous studies have investigated, however, the effects of BoNT-A in multiple forms of dystonia and spasticity. As shown below, the spectrum of findings is rather broad, which probably reflects the distinct aetiologies of dystonic and spastic movement disorders, differences among patient cohorts, as well as the diversity of imaging protocols. The studies in the following section are therefore discussed with a special emphasis on factors that potentially account for those inconsistencies.

#### 3.2.1. Dystonia

Since BoNT-A is the recommended first-line treatment for focal dystonia, many imaging studies on dystonia involve patients receiving regular BoNT-A injections. Most of them assessed brain activation at a single time point (e.g., [68,69,70,71,72]), either in the middle [70,72] or at the end of the 3-month treatment cycle [68,71]. Another study included patients with a history of BoNT-A treatment, but currently off treatment for several years [73]. Hence, only interventional studies involving at least two measurements (before and after BoNT-A) are further discussed in detail, with a few exceptions being substantiated by relevant findings. Notably, prominent differences may be observed even among the studies that evaluated BoNT-A effects using repeated examinations before and after treatment. These inconsistencies may arise from the long-term effects of BoNT-A involving neuronal plasticity and may differ from the short-term effects of the first dose [3,8,11,12]. For this reason, the distinction between BoNT-A-naïve and pre-treated cohorts has been taken into consideration in the following paragraphs. The functional neuroimaging studies assessing the central effects of BoNT-A in focal dystonias are also summarised in Table 2.

##### BoNT-A Effect on Somatosensory Task-Related Activation

As discussed in previous sections and demonstrated in multiple electrophysiological [37,38,39,41,90] and imaging studies [72,91,92] involving peripheral stimulation, dystonia is characterised by abnormal somatosensory processing and considered to be a disorder of sensorimotor integration [8]. Three studies have therefore evaluated the central effects of BoNT-A by comparing pre-treatment and post-treatment brain responses to external stimulation [74,75,76].

A study in pre-treated patients with blepharospasm and Meige’s syndrome compared somatosensory activations during tactile stimulation of the forehead, lips, and hand. Prior to BoNT-A, patients hypoactivated the bilateral S1 and right S2 (regardless of the stimulated side). This hypoactivation remained unchanged, however, after the treatment. In contrast, BoNT-A reduced activation in the left mesial premotor cortex (PMC), also known as the supplementary motor area (SMA), when the ipsilateral lips or the contralateral forehead and hand were stimulated. Furthermore, the treatment reduced activation in the bilateral thalami and contralateral putamen during the stimulation of the forehead on either side [74].

An fMRI study from our laboratory utilised electrical median nerve stimulation in patients with cervical dystonia who were regularly receiving BoNT-A [75]. In partial agreement with the conclusions of Dresel et al. [74], our study demonstrated that patients with cervical dystonia hypoactivated the contralateral S2 and insula at baseline, i.e., after the expiration of the previous BoNT-A injection. In this study, however, the hypoactivation was restored back to normal, 4 weeks after the BoNT-A injection [75].

A recent study by Mantel et al. [76] evaluated the effect of BoNT-A in 12 pre-treated patients with adductor-type spasmodic dysphonia using the tactile stimulation paradigm of Dresel et al. [74]. In contrast to the results of Dresel et al. [74], patients hyperactivated a number of cortical areas at baseline (pre-BoNT-A) [76]. The contralateral S1 and S2 were overactivated during the stimulation of the right forehead and lip, while the contralateral S1 was also overactivated during the stimulation of the left hand and upper lip. Further hyperactivated areas included: the contralateral posterior parietal cortex (intraparietal sulcus [IPS], superior parietal lobule [SPL]), insula, ventral PMC/M1 (during stimulation of the left lip only) and the left superior temporal gyrus. A comparison before and after BoNT-A, however, did not reveal any significant changes. The authors of the study suggested that the differences between their results and the results of Dresel et al. [74] are inherent to the differences between the task-specific and non-task-specific forms of dystonia [76].

The combined evidence from the three studies [74,75,76] therefore suggests that abnormal (reduced) sensory processing in the S2 could be a common feature of several forms of non-task-specific dystonia. BoNT-A treatment seems to have unique (if any) effects on somatosensory processing depending on the stimulation site and the underlying disease form. Although these changes in somatosensory system may have direct relationship to the effects induced by BoNT-A, further replications are needed to confirm this.

##### BoNT-A Effect on Motor Task-Related Activation

In addition to aberrant somatosensory processing, various forms of dystonia have also been associated with a range of abnormal findings during motor performance, including task-related hyperactivation [71,81,93], hypoactivation [69,78,82], or both [68,77,79,80,94]. Despite the high variability of results, there have been some recurring observations, including overactivation of the basal ganglia [71,94], cerebellum [68,77,79,80,93], anterior cingulate [79,93,94], lateral PMC [68,77,79,93,94] and parietal cortices [77,79,80,81,93], but also hypoactivation of the parietal cortices [68,78], basal ganglia [69,82], SMA [77,78,79,82,94], PMC [78,80], primary motor cortex (M1) [77,78,80,94] and anterior cingulate cortex [77,78,82]. Further abnormalities in patients with dystonia were documented during passive movements [70] and motor imagery [69,73], but a complete account of all the differences is beyond the scope of this review. The provided list illustrates, however, that it is currently impossible to delineate a single activation pattern associated with dystonia in general. It further indicates that it would be equally difficult to identify any universal pattern of changes following BoNT-A injections. Nevertheless, the following text attempts to provide a comprehensive overview of treatment-related activation changes to facilitate the search for common features that might be identified as specific effects of BoNT-A in the future.

Among studies directly assessing BoNT-A effects, two have utilised H_2_^15^O positron emission tomography (PET). A study by Ceballos-Baumann et al. [77] evaluated activation during writing in patients with writer’s cramp who were receiving chronic BoNT-A treatment but were off medication for more than 3 months. It revealed reduced activation in the contralateral M1 and SMA with simultaneously enhanced regional cerebral blood flow in the ipsilateral PMC, contralateral S1, bilateral posterior parietal cortices, and in the cerebellar vermis. In a follow-up examination, the treatment with BoNT-A had no effect on the hypoactivation in the M1, but instead, it further increased activation in the already overactivated contralateral S1, enhanced and normalised activation in the SMA, and reduced activation in the anterior cingulate and cerebellum. After treatment, patients consequently showed even stronger hyperactivation of the bilateral premotor and parietal cortices, expressed no differences in the cerebellum or SMA, but still hypoactivated the contralateral M1. It was suggested that the increased activation in parietal regions reflected the actual cortical reorganisation following BoNT-A [77].

The second H_2_^15^O PET study [79] showed that baseline activation during speech production in patients with spasmodic dysphonia was decreased in the SMA and increased in the cerebellum, similar to patients with focal hand dystonia in the previous study [77]. The speech-related activation was, however, additionally decreased in the right anterior temporal cortex, left temporoparietal cortex, S1, and brainstem, whereas it was increased in the left S2, right M1/PMC, insula, bilateral posterior temporal cortices, and anterior cingulate. Most of the included patients were chronically treated with BoNT-A (7 out of 9), but at the time of assessment, they were off medication for at least 6 months. In a follow-up PET after BoNT-A, activation increased in the left temporoparietal cortex and brainstem, which were originally attenuated in patients, and decreased in some areas that were originally hyperactivated, including the cerebellum, right M1/PMC, insula, left auditory cortex and anterior cingulate. No change was observed in the SMA proper; instead, BoNT-A not only enhanced activation in the left ventral M1/PMC, frontal operculum and insula but also lowered activation in the right thalamus and putamen, left caudate, and pre-SMA. The activation decreases in the cerebellum, anterior cingulate, and right thalamus as well as the increase in the left temporoparietal cortices, brainstem, and left frontal operculum were consistently correlated with clinical improvement [79].

Further research on the central effects of BoNT-A in dystonia was conducted using blood oxygenation level-dependent (BOLD) fMRI imaging. While most studies assessed activation during active movements [78,80,81,82], a single study involved passive movements [70], representing a transition between pure stimulation and motor-task protocols. The study by Obermann et al. [70] evaluated activation during passive forearm flexion in patients with cervical dystonia chronically treated with BoNT-A. The activation in patients was increased in the contralateral S1, S2/insula, bilateral cingulate cortex and cerebellum in the middle of the 3-month treatment cycle. Although only one time point was evaluated, a positive correlation with the applied dose of BoNT-A and a negative correlation with the Toronto Western Spasmodic Torticollis Rating Scale (TWSTRS) score was detected in the SMA. In other words, the higher the activation in the SMA, the higher the applied dose of BoNT-A and the lower the clinical severity after treatment.

The remaining fMRI studies involved active motor performance, either related or unrelated to dystonic manifestation. Haslinger et al. [78] assessed vocalisation and whispering in pre-treated patients with spasmodic dysphonia. At baseline, activation was attenuated in the bilateral primary sensorimotor cortex (SMC), anterior cingulate, SMA, dorsal PMC, and sensory association cortices. Subsequent BoNT-A treatment had no effect, however, on these abnormalities. In the second study by the same group [80], activation was evaluated during whistling in mostly pre-treated patients with blepharospasm and Meige’s syndrome. Both patient groups overactivated the bilateral S1 and SMA before treatment, although the hyperactivation of the SMA was only observed at a lower statistical threshold in patients with Meige’s syndrome. Baseline activation associated with pure blepharospasm was also increased in the left dorsolateral prefrontal cortex (DLPFC) and either increased or decreased in different parts of the left cerebellum, whereas in Meige’s syndrome, baseline activation was additionally reduced in the bilateral M1 and ventral PMC. After treatment, activation decreased in the left SMA and right parietal cortex (S1 and inferior parietal lobule [IPL]) in the Meige’s syndrome group, although there was no change in the blepharospasm group [80].

Two fMRI studies from our lab evaluated activation in patients with cervical dystonia performing sequential finger opposition (SFO) [81,82]. In the first study, Opavský et al. [81] demonstrated that patients receiving BoNT-A treatment hyperactivated the contralateral S2 at the time of their next scheduled BoNT-A injection. Application of BoNT-A led to activation decrease in the SMA and dorsal PMC 4 weeks later. At that point, patients hypoactivated the bilateral pallidum compared to healthy subjects. A follow-up study by Nevrlý et al. [82] in a cohort of BoNT-A-naïve patients revealed that baseline performance of the same motor task was associated more with hypoactivation of the bilateral SMA, cingulate and paracingulate cortices and the ipsilateral caudate, pallidum and thalamus. Application of BoNT-A resulted in a wide-spread activation increase throughout the sensorimotor cortices, including the bilateral dorsal PMC, SMA, anterior cingulate cortex, S1, S2, insulae, posterior parietal cortices, and contralateral M1. In addition, activation increased in several mostly ipsilateral subcortical areas, including the thalamus, putamen, midbrain and ipsilateral cerebellar hemisphere and vermis [82]. The apparently opposite changes in the SMA and dorsal PMC observed by Opavský et al. [81] were thought to reflect plastic changes in long-term treated patients [82]. These shifts in motor cortex responses to BoNT-A treatment may also explain some inconsistencies among the previous imaging studies. While most of the studies evaluating BoNT-A intervention in task-related fMRI included patients already on regular treatment [74,75,77,78,81], some actually involved mixed cohorts of treated and untreated patients [79,80]. The study from our group [82] has provided thus far the only evidence of BoNT-A effects on task-related activation in naïve patients with dystonia.

In summary, application of BoNT-A in pre-treated patients with dystonia was associated with all possible outcomes, including task-related activation increase [77], decrease [80,81], both [79], or none [78]. The localisation of the reported differences varies considerably, including increased activation in the parietal cortices [77,79], M1/PMC [79], SMA [77], insula and brainstem [79], but also reduced activation in the parietal cortices [80], M1/PMC [79,81], SMA [80,81], anterior cingulate [77,79], insula, thalamus and basal ganglia [79], and cerebellum [77,79]. In contrast, BoNT-A naïve patients expressed only large activation increases after treatment, including the parietal cortices, M1/PMC, SMA, anterior cingulate cortex, insulae, basal ganglia, brainstem, and cerebellum [82]. Therefore, the data available thus far only indicate that treatment with BoNT-A is associated with distributed sensorimotor adaptations, but the directionality and localisation of changes differ depending on the imaging procedure (task), dystonic phenotype, and the previous treatment. While some intersections among the results are apparent, they are far from being the basis for consensus. The current state of evidence requires further confirmation in better characterised and larger patient cohorts, as well as clear-cut outcome measures that can be associated with activation changes.

##### BoNT-A Effect on Resting-State Connectivity

Additional insight into the central effects of BoNT-A may be obtained by analysing data at rest, which are unaffected by the a priori selected stimulation or task. While multiple studies evaluated resting state brain function in dystonia to determine disease traits (e.g., [95,96,97]), this section focuses on studies specifically evaluating the effects of BoNT-A intervention. A fluoro-deoxy-glucose PET study [83] evaluated resting state metabolism in patients with blepharospasm at a single time point following BoNT-A injections. Patients were divided into two groups, either showing a complete or incomplete treatment response. The results revealed that, regardless of the treatment outcome, blepharospasm was associated with increased resting metabolism in the bilateral thalami and pons. Uncorrected maps additionally suggested that patients with incomplete improvement had increased glucose metabolism in the cerebellum, but the group difference was not significant [83]. The same group later described a similar increase of resting glucose metabolism in the bilateral thalami in non-dystonic abnormal movements, namely in hemifacial spasm [98]. This indicates that resting hyperactivaction of the thalami might be secondary to abnormal movements rather than a causal pathophysiological phenomenon.

Alteration of the resting-state brain function has more often been evaluated using BOLD fMRI than PET. While PET involves relevant baseline information about cerebral blood flow or metabolism, BOLD fMRI generates dimensionless data that cannot be directly compared between groups of subjects. Instead, fMRI data have frequently been utilised to evaluate correlations in spontaneous BOLD signal fluctuations (functional connectivity, FC) or even more complex metrics describing either local signal features (e.g., regional homogeneity, ReHo) or global network-wide properties. A study by Mohammadi et al. [84] evaluated, for example, resting-state FC using independent component decomposition in 16 patients with writer’s cramp on chronic BoNT-A therapy in comparison with healthy subjects. They observed increased FC between the default mode network and the contralateral putamen, and decreased FC between the bilateral sensorimotor network and the S1 contralateral to the symptomatic right hand. None of these differences were affected, however, by the subsequent BoNT-A application.

Another study using independent component analysis (ICA) in pre-treated patients with cervical dystonia by Delnooz et al. [85] revealed baseline abnormalities in three large-scale networks in comparison with healthy controls. The observed differences involved decreased FC between the sensorimotor network (consisting of the PMC, SMA, primary SMC, and S2) and prefrontal cortex, PMC, and SPL, as well as decreased FC between the (primary) visual network and the prefrontal cortex, PMC, SPL, and middle temporal gyrus. FC also increased between the executive control network (consisting of the anterior cingulate, prefrontal, and parietal cortices) and the M1, PMC, prefrontal and visual cortices. Application of BoNT-A led to partial normalisation of the abnormal connectivity of the sensorimotor and visual networks. Namely, FC increased between the primary visual network and the M1 and secondary visual cortex, as well as between the sensorimotor network and the ventral PMC. Notably, FC between the ventral PMC and the sensorimotor network decreased again at the third time point 3 months after the BoNT-A injection [85]. In a follow-up study by the same group [86], the authors examined the voxel-wise connectivity of the basal ganglia in a similar cohort of pre-treated patients with cervical dystonia. They reported reduced FC between the left (associative) frontoparietal network and the right putamen. The same network also showed a trend towards decreased connectivity with the right external pallidum, while a trend towards increased FC was reported between the bilateral putamina and the sensorimotor network (neither being significant after Bonferroni correction). The BoNT-A treatment influenced different connections, however, as it enhanced FC between the executive control network and the right ventral striatum and external pallidum [86].

Resting state connectivity was also studied in patients with blepharospasm and Meige’s syndrome [87]. In a cohort of regularly treated patients, baseline (off BoNT-A) connectivity was abnormally reduced among the multiple cortical and subcortical regions, including (1) FC between the caudate nucleus and the primary SMC, parietal and visual cortices; (2) FC between the putamen and the temporal and parietal cortices; (3) FC between the cingulate cortex, and the primary SMC, PMC and parietal cortices; (4) FC between the PMC and the S1; and (5) FC between the S1 and the S2, cingulate cortex and cerebellum. The cerebellum also showed decreased connectivity with visual cortices, which was the only connection that was augmented after BoNT-A. In contrast, several areas exhibited further decreases in FC after treatment. Connectivity strength was reduced (1) between the pallidum and the cerebellum, caudate nucleus, and putamen; (2) between the cerebellum and the posterior cingulate cortex, prefrontal, parietal, temporal, visual, premotor cortices, and SMA; and (3) between the thalamus and the SMA/cingulate cortex [87]. The increased post-BoNT-A connectivity from the cerebellum supports its specific role in the pathophysiology of dystonia [99,100,101], in line with previous reports of abnormal cerebellar resting-state connectivity [95,96] and task-related activation [102].

Recently, resting-state connectivity was also evaluated in 12 patients with adductor-type spasmodic dysphonia receiving chronic BoNT-A treatment [76]. In this study, baseline analysis of differences between patients and controls revealed several abnormalities in FC (increased FC between the right SMC and the rest of the sensorimotor network, as well as increased FC between the left auditory cortex and the rest of the auditory network) and ReHo (reduced ReHo in the right temporoparietal junction. No changes, however, after BoNT-A were detected.

The effects of BoNT-A might be essentially different in patients who were never exposed to BoNT-A before. A study by Brodoehl et al. [88] investigated multiple resting-state parameters in 17 patients with cervical dystonia naïve to BoNT-A treatment. The baseline comparison specifically showed decreased FC between the S1 and S2, as well as increased FC among the basal ganglia, and between the thalamus and basal ganglia. The second assessment was scheduled 2 weeks after the third BoNT-A injection (administered every 3 months). At this point, the baseline group differences in FC partially reverted and the connectivity patterns of patients and control subjects converged. Using Granger causality and Granger autonomy, the authors further evaluated effective connectivity and the independent sources of the neuronal signal. The baseline data indicated that the putamen, thalamus and S1 were increasingly independent in patients and that the putamen influenced other regions, including the S2, which further influenced the M1. After treatment, the information flow from the putamen and thalamus into the S2 was reduced; instead, the influence of the M1 increased in the caudate nucleus. In the final analysis, the authors reported that baseline ReHo was significantly increased in the M1, S1, putamen and thalamus. Among these, ReHo decreased after treatment in the putamen and S1. The study thus reported consistent abnormalities characterised by deficient somatosensory integration between the S1 and S2, and by increased intrinsic connectivity within the cortico-subcortical loops driven by subcortical (putaminal and thalamic) sources that partially normalised after treatment. There was no correlation, however with TWSTRS scores in any parameter, which gave rise to further questions regarding the relationship between these findings and the symptom severity. The study was also a priori limited to a rather small set of cortical and subcortical regions of interest (ROI) and did not evaluate, for instance, the involvement of the cerebellum [88].

Our recent efforts to elucidate the role of the cerebellum in mediating the effects of BoNT-A in a cohort of 17 naïve patients [89] indicate that cortico-cerebellar connectivity is significantly affected by the treatment in several areas: on average, treatment reduced FC between the vermis lobule VIIIa and the left dorsal mesial frontal cortex. In addition, reduction in FC between the nearby vermis lobule VIIIb and the bilateral prefrontal cortices and right temporoparietal junction was positively correlated with improvement in clinical scores. The same correlation was observed for the FC between the right crus II and the ipsilateral prefrontal cortex. Additionally, similar positive correlations were observed for intracerebellar connectivity between the anterior (right VI) and the posterior (right crus II) cerebellum, as well as between the right lobule IX and the left lobule VI–VII [89].

In summary, the changes in resting-state connectivity occurring after BoNT-A application are as manifold as are the observations in task-related studies. While resting-state PET studies indicated changes in thalamic activation [83,98], fMRI studies pointed to more wide-spread effects including changes in intracortical [85,88], cortico-subcortical [86,87,88], cortico-cerebellar [87,89], striato-cerebellar, pallido-cerebellar [87], and intracerebellar connectivity [89]. Nevertheless, negative results (i.e., no treatment-related changes) were also reported [76,84]. While such a variety of results certainly indicates the far-reaching effects of BoNT-A, it is challenging to identify a single key structure or cortical area that would be responsible for all the observed changes. In fact, these data tend to support the notion that dystonia is a network-wide disorder in which a lesion of any single node could lead to a common manifestation [82,103,104]. Similarly, the central effects of BoNT-A may hinge upon dynamic modulatory changes in multiple nodes of the sensorimotor network, which could be differently weighted in various patient cohorts, explaining both the variability of clinical manifestations and the individual responses to treatment. The network-wide hypothesis of pathophysiology of dystonia has recently been challenged, however, as more focal differences in FC have been reported in the striatum and sensorimotor cortex after more rigorous approaches had been applied [97]. Hence, regionally specific effects of BoNT-A are plausible if similar rigorous methods are used. Studies with larger samples and reliable denoising procedures are needed to confirm this.

#### 3.2.2. Spasticity

As opposed to relatively scarce electrophysiological evidence, there has been an increasing number of imaging studies assessing the central effect of BoNT-A in spasticity. Since spasticity is a common consequence of stroke [105], most of the research has been dedicated to stroke patients, whereas studies in other patient cohorts are less frequent. For the same reason, the effects of BoNT-A have been most frequently investigated in patients with upper limb spasticity. Importantly, comprehensive treatment in stroke patients also requires regular physiotherapy, therefore, the reported effects of BoNT-A are usually combined with the effects of physiotherapy [106]. As this is the recommended treatment approach, application of BoNT-A without physiotherapy would be unethical and, therefore, their effects have usually been studied together [107,108,109,110,111,112,113] but see [114,115]. Despite a wide range of structural lesions that lead to spasticity, the following paragraphs illustrate that changes observed in spasticity seem to be much more uniform than observations in dystonia.

A study by Bergfeldt et al. [107] in 6 chronic stroke patients used finger extension-flexion to investigate motor task-related activity before the BoNT-A injection and at two follow-up sessions after 6 and 12 weeks. Using a ROI-based analysis of individual BOLD responses rather than group-wise statistics, the authors demonstrated increased activation levels in patients in the contralesional M1/PMC with reduced lateralisation of activation as compared to controls. As spasticity improved after BoNT-A, activation levels decreased numerically in both ipsilesional and contralesional cortices after treatment. Since a larger change was detected in the ipsilesional cortex, the treatment partially normalised the lateralisation of cortical activation. At the second follow-up, activation increased numerically, but overall, it remained lower than at baseline. The within-group differences were not formally statistically tested, however, calling into question the statistical significance of the observed differences [107]. The results are nevertheless in line with those observed by Manganotti et al. [115] who utilised combined EMG-fMRI imaging in 8 chronic stroke patients naïve to BoNT-A during an isotonic hand grip task. Before BoNT-A, patients activated a bilateral network of areas consisting of the primary SMC, SMA and the cerebellum. Using an ROI-based approach, this study revealed that the extent of activation (number of active voxels) decreased bilaterally and the distribution of active voxels was more lateralised than at baseline. Importantly, EMG recordings showed no muscle activity in the contralateral hand at any time point, instead, they illustrated a reduction of co-contractions in the paretic hand [115]. Another small study in 4 chronic stroke patients using a similar task showed an overactivation in the cerebellum during gripping with the paretic hand; however, there were no significant changes 1 week after injection, possibly due to small sample size and too short follow-up [114].

The effects of BoNT-A on brain activations in patients with spasticity have also been extensively evaluated in a large series of fMRI studies from our lab [108,109,110,111,112,113,116,117]. In several studies on upper limb spasticity, our lab has utilised complex SFO [109,111,113] according to Roland et al. [118]. In patients with hand paralysis who were not able to perform active movements, we utilised passive hand movements [112] and kinaesthetic movement imagery [108,110,111]. All patients included in the studies were naïve to the BoNT-A treatment and all received concomitant physiotherapy.

The feasibility of movement imagery as a substitute for real movements in assessment of the central effects of BoNT-A was demonstrated by Šenkárová et al. [108] in a preliminary study including 4 hemiplegic patients. The task involved performance of kinesthetic imagery of complex SFO using the plegic hand after training the same movement with the unaffected hand. A comparison of BOLD activations before and 4 weeks after BoNT-A showed a significant decrease in activation in the posterior cingulate cortex. The average activation maps also indicated a global decrease of activation throughout the sensorimotor system [108]. These findings were further expanded by a follow-up study by Veverka et al. [110] that utilised the same task in 14 patients following a longitudinal design with examination scheduled before BoNT-A and 4- and 11-weeks post-treatment. Group-wise maps again showed an overall reduction of the activation extent, which continued throughout the follow-up. Direct contrasts confirmed decreased activation in the posterior parietal cortex (IPL and precuneus). At the final follow-up, activation further decreased in the bilateral prefrontal cortices and ipsilesional insular cortex. The differences were the most extensive when the first examination was contrasted with the final one when they could also be observed in the contralesional primary SMC [110].

In patients with severe hand paresis, the effect of BoNT-A was also assessed using passive wrist movements [112]. The study in 7 hemiplegic patients followed the same longitudinal design with a baseline exam before treatment and re-evaluation at 4- and 11-weeks post-treatment. In contrast to the active movement imagery, application of BoNT-A resulted in an activation increase in the bilateral posterior cerebellum and occipital cortices. At the second follow-up, activation decreased in the anterior cerebellum and SMA/pre-SMA. The decrease in the SMA, along with the reduced activation in the ipsilesional primary SMC (foot area), was also significant when compared to the study baseline. While it may seem that BoNT-A effects in kinesthetic imagery [108,110] and passive movements [112] are contradictory, it was argued that BoNT-A may have an essentially distinct influence on internally driven and externally evoked activation. It was further suggested that reduced abnormal (noisy) afferentation evoked implicit motor visualisation [112].

In patients with less severe hand paresis, use of overt active movements allowed for a more direct investigation of the central influence of BoNT-A on motor control. In a group of 5 hemiparetic patients after stroke, Tomášová (formerly Šenkárová) et al. [109] utilised SFO to assess longitudinal changes in brain activation following BoNT-A. The study showed that, 4 weeks after BoNT-A application, the extent of group-wise activation was apparently reduced, but it returned to the original state at week 11. Although a direct comparison revealed no significant voxel-wise differences, a weighted contrast between session 2 and sessions 1 and 3 revealed a treatment-related activation decrease in the ipsilesional inferior frontal gyrus, DLPFC, dorsal PMC, postcentral gyrus and IPL, representing the transient effect of BoNT-A controlled for the effect of concomitant physiotherapy [109].

In another study [111], BoNT-A effects on real and imagined movements were more closely compared in two groups of patients matched for age (7 patients per group). In the plegic group performing kinaesthetic imagery, activation transiently decreased in the posterior cingulate and occipital cortices 4 weeks after BoNT-A and increased again at 11 weeks post-treatment. In the paretic group performing overt SFO, activation extensively decreased throughout the sensorimotor system, predominantly in the ipsilesional DLPFC, dorsal PMC, SMA, primary SMC (foot area) and posterior parietal cortex (SPL and IPL), but also in the bilateral inferior frontal, orbitofrontal and occipital cortices. At the final follow-up, activation increased again in a subset of these areas, namely in the anterior cingulate, ipsilesional posterior parietal (IPL, SPL) and inferior frontal cortices. In contrast, activation remained reduced in the bilateral occipital cortices [111].

Our most recent and largest study thus far evaluated 30 patients with post-stroke spasticity and mild paresis [113]. It again followed the same longitudinal design and analysis aimed at disentangling the effects of BoNT-A from the effects of concomitant physiotherapy using three time points and a weighted contrast. It demonstrated that the central cortical structure reflecting the transient improvement of spasticity was localised to the ipsilesional posterior parietal cortex (SPL and IPS) which decreased transiently after BoNT-A. No consistent effect of time (on physiotherapy) was observed. This result is in line with our previous reports, where a decrease in posterior parietal activation was consistently observed [109,111], including kinaesthetic motor imagery data [110]. While differences in other cortical areas are likely to accompany the changes in parietal cortices, modulation of the ipsilesional SPL/IPS seems to be the least variable change. Further studies are warranted, however, in order to establish whether the activation decrease in SPL/IPS is simply a marker BoNT-A effect or has a causal relationship to the clinical improvement.

Diserens et al. [119] used a different paradigm to study the effect of combined BoNT-A and rehabilitation in post-stroke arm spasticity patients. Namely, they evaluated the effect of BoNT-A application with or without add-on three-month repetitive arm cycling, with fMRI examinations at baseline and after 3 months. This design did not allow for separation of the transient BoNT-A effect, which expires within 3 months, from the long-term effect of rehabilitation. The significant clinical improvement of spasticity in a subgroup of patients with residual motor activity was accompanied by BOLD activity increases in the ipsilesional primary sensorimotor cortex and in the contralesional secondary somatosensory area.

Apart from evidence from chronic stroke patients, our preliminary fMRI study assessed activation changes following BoNT-A in 4 multiple sclerosis patients with lower limb spasticity and 4 control subjects [116]. In the study, patients received their first-time BoNT-A into the spastic hip adductor muscles. During the fMRI acquisition, they performed extension-flexion of the knee. The examinations were scheduled immediately before BoNT-A as well as 4 and 12 weeks after injection. In general agreement with data on post-stroke spasticity, patients showed overactivation in the bilateral sensorimotor cortices (mostly dorsal PMC and SPL) at baseline, which was reduced to a normal level after BoNT-A, but returned close to the original state at week 12 when the parietal cortex was again hyperactivated by patients relative to controls. This illustrates that the effects of BoNT-A on spasticity are likely to have more universal impact on brain activation, independent on injection site and underlying aetiology of spasticity.

#### 3.2.3. Summary of the Central Effects of BoNT-A

As illustrated in the previous paragraphs, muscle denervation using BoNT-A has a considerable impact on the function of the CNS structures. The most consistent findings include imaging reports of decreased sensorimotor activation during voluntary movements and kinaesthetic imagery in post-stroke spasticity, with possibly the central role of the ipsilesional SPL/IPS. Widespread activation changes were also observed in patients with dystonia, however, the individual patterns of changes seem to differ considerably among patient cohorts, potentially reflecting different underlying aetiologies, but also the variety of imaging protocols and methods of statistical analysis. Electrophysiological evidence for the central effects of BoNT-A was also reported, but the amount of literature is scarce, especially in spasticity. For a complete understanding of the central effects of BoNT-A, studies with healthy subjects are also desirable, although they are certainly more controversial to conduct. Moreover, to establish any causal relationship between clinical improvement and the central effects of BoNT-A, specific interventions should be designed that would either mitigate or augment the clinical effects by interaction with the putative central targets of BoNT-A.

## Figures and Tables

**Table 1:** Overview of electrophysiological and neuroimaging techniques referred to in the review (adapted from Di Pino et al. [15] and Matthews [16]).

**Electrophysiological Methods to Evaluate Plasticity** [15]				
**Abbreviation**	**Full Term**	**Technique**	**Anatomical/Physiological Substrate**	**Effect**
CSP	cortical silent period	suprathreshold TMS pulse delivered during tonic contralateral muscle contraction	intracortical inhibitory circuitry	inhibitory
ICF	intracortical facilitation	subthreshold conditioning TMS pulse followed by suprathreshold pulse (ISI 8–30 ms)	intracortical excitatory circuitry (possibly NMDA glutamatergic [17])	facilitatory
IHI	interhemispheric inhibition	ipsilateral subthreshold conditioning TMS pulse followed by contralateral suprathreshold pulse (ISI 10–40 ms)	transcallosal inhibitory connections	inhibitory
MEP	motor evoked potential or M-wave	see TMS		
P22/N30	complex of short-latency positive and negative waves	cortical responses to stimulation of the contralateral median nerve recorded precentrally	possibly activation of SMA and dorsolateral frontal cortex [18]	N/A
SEP	somatosensory evoked potentials	electrical stimulation of a peripheral nerve (usually median or tibial)	dorsal column–medial lemniscus pathway	spinal and cortical responses
SICI	short-interval intracortical inhibition	subthreshold conditioning TMS pulse followed by suprathreshold pulse (ISI 1–5 ms)	intracortical inhibitory circuitry (GABA-ergic [19])	inhibitory
TMS	transcranial magnetic stimulation	strong magnetic pulse delivered via round or figure-of-eight coil	transsynaptic activation of the corticospinal neurons in M1	suprathreshold stimulus triggers MEP (usually the test stimulus), subthreshold stimuli have conditioning effects
**Neuroimaging methods to evaluate plasticity** [16]				
**Abbreviation**	**Full term**	**Technique**	**Anatomical/physiological substrate**	**Modalities**
BOLD	blood oxygenation level-dependent	fMRI technique utilizing T_2_^*^ sequences sensitive to local field inhomogeneities associated with the blood oxygenation level	neuronal activation, signal convolved with hemodynamic response function due to neurovascular coupling delay	task-related, resting-state
fMRI	functional magnetic resonance imaging	usually BOLD fMRI, infrequently arterial spin labelling	see BOLD	
PET	positron emission tomography	H_2_^15^O PET, fluoro-deoxy-glucose PET or similar	based on the radiotracer, usually reflecting regional cerebral blood flow or neuronal metabolism	task-related, resting-state
**Methods to experimentally induce plasticity** [15]				
**Abbreviation**	**Full term**	**Technique**	**Anatomical/physiological substrate**	**Effect**
PAS	paired associative stimulation	peripheral electric stimulus time-locked to a TMS pulse at the M1	intracortical and thalamocortical circuits	based on ISI
rTMS	repetitive TMS	suprathreshold repetitive TMS pulses	LTP-like or LTD-like corticospinal plasticity	excitatory (high frequency) or inhibitory (low frequency)

Abbreviations (abbreviations appearing only in the first column are explained in the table): fMRI—functional magnetic resonance imaging, GABA—γ-aminobutyric acid, ISI—interstimulus interval, LTD—long-term depression, LTP—long-term potentiation, M1—primary motor cortex, MEP—motor evoked potential, MRI—magnetic resonance imaging, N/A—not applicable, NMDA—N-methyl-D-aspartate, PET—positron emission tomography, SMA—supplementary motor area, TMS—transcranial magnetic stimulation.

**Table 2:** Chronological overview of functional neuroimaging studies of the central effects of botulinum neurotoxin type A (BoNT-A) in dystonia.

Authors	Disease	Cohort	Imaging Technique	Task/Analysis	Effect of BoNT-A
**Somatosensory Task-Related Activation**					
Dresel et al. [74]	blepharospasm and Meige’s syndrome	16 pre-treated (10 with Meige’s syndrome)	fMRI	tactile stimulation of the forehead, lips, and hand	reduced activation in the left SMA, bilateral thalami and contralateral putamen
Opavský et al. [75]	cervical dystonia	7 pre-treated	fMRI	electrical median nerve stimulation	restored hypoactivation in the contralateral S2 back to normal
Mantel et al. [76]	adductor-type spasmodic dysphonia	12 pre-treated	fMRI	tactile stimulation of the forehead, lips, and hand [74]	no changes after BoNT-A
**Motor task-related activation**					
Ceballos-Baumann et al. [77]	writer’s cramp	6 (5 pre-treated and off BoNT-A for 4-12 M, 1 naïve)	H_2_^15^O PET	writing	increased activation in the already overactivated contralateral S1, enhanced and normalised activation in the SMA, and reduced activation in the anterior cingulate and cerebellum
Haslinger et al. [78]	spasmodic dysphonia	12 pre-treated patients	fMRI	vocalisation and whispering	no changes after BoNT-A
Ali et al. [79]	spasmodic dysphonia	9 (7 pre-treated and off BoNT-A for >6 M, 2 naïve)	H_2_^15^O PET	speech production	increased activation in the left temporoparietal cortex and brainstem (originally attenuated); decreased activation in the cerebellum, right M1/PMC, insula, left auditory cortex and anterior cingulate (originally hyperactivated); additionally enhanced activation in the left ventral M1/PMC, frontal operculum and insula; and lowered activation in the right thalamus and putamen, left caudate, and pre-SMA, some changes correlated with clinical improvement
Dresel et al. [80]	blepharospasm and Meige’s syndrome	13 with blepharospasm (2 naïve), 13 pre-treated with Meige’s syndrome	fMRI	whistling	decreased activation in the left SMA, right S1 and IPL in Meige’s syndrome group, no change in blepharospasm group
Obermann et al. [70]	cervical dystonia	17 pre-treated	fMRI	passive forearm flexion	positive correlation with the applied dose of BoNT-A and a negative correlation with TWSTRS in the SMA
Opavský et al. [81]	cervical dystonia	7 pre-treated	fMRI	sequential finger opposition	decreased activation in the SMA and dorsal PMC
Nevrlý et al. [82]	cervical dystonia	12 naïve	fMRI	sequential finger opposition [81]	increased activation in the bilateral dorsal PMC, SMA, anterior cingulate cortex, S1, S2, insulae, posterior parietal cortices, contralateral M1, mostly ipsilateral thalamus, putamen, midbrain and ipsilateral cerebellar hemisphere and vermis
**Resting-state connectivity**					
Suzuki et al. [83]	blepharospasm	25 with unknown treatment status	FDG PET	resting state metabolism	no significant difference between full responders and partial responders
Mohammadi et al. [84]	writer’s cramp	16 pre-treated	fMRI	independent component analysis	no changes after BoNT-A
Delnooz et al. [85]	cervical dystonia	23 pre-treated	fMRI	FC using dual regression with pre-defined RSN maps	increased FC of the primary visual network with the M1 and secondary visual cortex, and increased FC of the sensorimotor network with the ventral PMC
Delnooz et al. [86]	cervical dystonia	23 pre-treated	fMRI	voxel-wise FC of the basal ganglia with pre-defined RSN	increased FC of the executive control network with the right ventral striatum and external pallidum
Jochim et al. [87]	blepharospasm and Meige’s syndrome	13 pre-treated (4 with Meige’s syndrome)	fMRI	seed-based FC of 45 atlas-based cortical, subcortical and cerebellar ROIs	increased FC of the cerebellum with visual cortices (originally decreased), decreased FC of the pallidum with the cerebellum, caudate nucleus, and putamen; decreased FC of the cerebellum with the posterior cingulate cortex, prefrontal, parietal, temporal, visual, premotor cortices, and SMA, and decreased FC of the thalamus with the SMA/cingulate cortex
Brodoehl et al. [88]	cervical dystonia	17 naïve	fMRI	seed-based FC of 18 atlas-based cortical and subcortical ROIs; Granger causality and Granger autonomy; ReHo	increased FC between S1 and S2, decreased FC within the basal ganglia and between the basal ganglia and thalamus or cortex, i.e., partial normalisation of FC; reduced information flow from the putamen and thalamus into the S2, increased influence of the M1 in the caudate nucleus; decreased ReHo in the putamen and S1; no correlation with TWSTRS
Mantel et al. [76]	adductor-type spasmodic dysphonia	12 pre-treated	fMRI	FC using independent component analysis, ReHo	no changes after BoNT-A
Hok et al. [89]	cervical dystonia	17 naïve	fMRI	seed-based FC from 26 atlas-based ROIs in the cerebellum	reduced FC of the vermis lobule VIIIa with the left dorsal mesial frontal cortex, correlation between the TWSTRS change and reduction in intracerebellar FC of the right VI and right lobule IX, and FC of the vermis lobule VIIIb and right crus II with the bilateral prefrontal cortices and right temporoparietal junction

Abbreviations: FC—functional connectivity, FDG—fluorodeoxyglucose, fMRI—functional magnetic resonance imaging, IPL—inferior parietal lobule, M—month(s); M1—primary motor cortex, PET—positron emission tomography, PMC—premotor cortex, ReHo—regional homogeneity, RSN—resting state network, S1—primary somatosensory cortex, S2—secondary somatosensory cortex, SMA—supplementary motor area, TWSTRS—Toronto Western Spasmodic Torticollis Rating Scale.
